# Targeting inflammasome/IL-1 pathways for cancer immunotherapy

**DOI:** 10.1038/srep36107

**Published:** 2016-10-27

**Authors:** Beichu Guo, Shunjun Fu, Jinyu Zhang, Bei Liu, Zihai Li

**Affiliations:** 1 Department of Microbiology and Immunology, Medical University of South Carolina, 173 Ashley Avenue, Charleston, SC 29425, United States of America; 2 Hollings Cancer Center, Medical University of South Carolina (MUSC), Charleston, South Carolina 29425-5040. United States of America

## Abstract

The inflammatory microenvironment has been shown to play important roles in various stages of tumor development including initiation, growth, and metastasis. The inflammasome is a critical innate immune pathway for the production of active IL-1β, a potent inflammatory cytokine. Although inflammasomes are essential for host defense against pathogens and contribute to autoimmune diseases, their role in tumor progression remains controversial. Here, our results demonstrate that the inflammasome and IL-1β pathway promoted tumor growth and metastasis in animal and human breast cancer models. We found that tumor progression was associated with the activation of inflammasome and elevated levels of IL-1β at primary and metastatic sites. Mice deficient for inflammasome components exhibited significantly reduced tumor growth and lung metastasis. Furthermore, inflammasome activation promoted the infiltration of myeloid cells such as myeloid-derived suppressor cells (MDSCs) and tumor-associated macrophages (TAMs) into tumor microenvironments. Importantly, blocking IL-1R with IL-1R antagonist (IL-Ra) inhibited tumor growth and metastasis accompanied by decreased myeloid cell accumulation. Our results suggest that targeting the inflammasome/IL-1 pathway in tumor microenvironments may provide a novel approach for the treatment of cancer.

Accumulating data indicate that tumor development not only depends on genetic alternations within malignant or premalignant cells, but also on the inflammatory microenvironment^1^,^2^,^3^,^4^,^5^. However, innate pathways regulating the inflammatory response in tumor microenvironments are not fully understood. The inflammasome is an important innate immune pathway responsible for the production of active IL-1β, and induction of pyropoptosis^6^,^7^,^8^,^9^,^10^. While inflammasomes are critical for the immune response against infections, and the development of certain autoimmune diseases^9^,^10^,^11^,^12^, the role of inflammasomes in tumor development remains poorly characterized.

An inflammasome is a multimolecular complex, composed of an NOD-like protein (NLR), the adaptor apoptosis-associated speck-like protein containing a caspase recruitment domain (ASC), and caspase-1, which is responsible for the cleavage of pro-IL-1β and pro-IL-18 proteins into their active forms. Therefore, production of mature or active IL-1β is controlled by at least two molecular mechanisms: first, Toll-like receptor (TLR) ligands or endogenous danger signals induce the expression of pro-IL-1β mRNA and proteins; the second signal triggered by very diverse stimuli activates inflammasomes, leading to IL-1β maturation and secretion. The NOD-like receptor family, pyrin domain containing 3 (NLRP3) inflammasome is the most studied one in this group, but other inflammasomes, including NLRP1, NLRC4, and absent in melanoma 2 (AIM2) inflammasomes, have also been identified^8^,^12^,^13^,^14^,^15^,^16^,^17^. Although many stimuli with very different and unrelated molecular structures induce the activation of the NLRP3 inflammasome, signal mechanisms leading to inflammasome activation remain elusive^13^,^18^.

While increased concentration of IL-1β protein in tumor tissues is associated with poor prognosis for cancer patients^19^,^20^,^21^,^22^,^23^, the function of inflammasomes in tumor growth and metastasis remains controversial. Published studies mainly use AOM/DSS-induced colon cancer as an animal model to study the involvement of inflammasomes in cancer. Results from those studies indicate that inflammasome components provide protections against tumorigenesis in colitis-associated colon cancer, as mice deficient for inflammasomes, including NLRP3, NLRP12, NLRC4 and caspase-1, have increased tumorigenesis in the AOM/DSS-induced colon cancer animal model^24^,^25^,^26^,^27^,^28^,^29^. However, in other types of cancer, such as melanoma and mesothelioma, inflammasomes and IL-1β have been shown to enhance tumor growth. In addition, chemotherapy drugs have been reported to induce inflammasome activation in mice with implanted tumors, which could either increase or decrease host anti-tumor immunity^15^,^30^,^31^.

In the immune response to pathogen infections, myeloid cells, particular macrophages, are a major cell source of inflammasome activation and IL-1β production^13^,^32^,^33^,^34^,^35^. Myeloid cells are an important component of the tumor microenvironments, and have been implicated in tumor growth and progression, as well as poor prognosis of cancer^36^,^37^,^38^,^39^. Myeloid cells infiltrated in tumor tissues are heterogeneous populations, primarily CD11b^+^Gr-1^+^ granulocytes, also referred to as myeloid-derived suppressor cells (MDSCs), and CD11b^+^F4/80^+^ Gr-1^−/low^ tumor-associated macrophages (TAMs)^38^,^40^,^41^,^42^,^43^,^44^,^45^,^46^,^47^. Those CD11b^+^ cells are present in the bone marrow and low levels in peripheral lymphoid organs of a normal host, but increase significantly in tumor tissues of tumor-bearing mice or human cancer patients. Thus, we hypothesized that myeloid cells contribute to tumor-associated inflammation and tumor progression through the production of IL-1β and other inflammatory mediators. Our results have demonstrated that inflammasome and IL-1β play a critical role in promoting tumor growth and metastasis. Furthermore, blocking IL-1R with IL-1R antagonist (IL-Ra) inhibits tumor progression accompanied by decreased myeloid cell recruitment in preclinical breast cancer models.

## Results

### Inflammasome promotes tumor growth and metastasis

**Figure 1:**
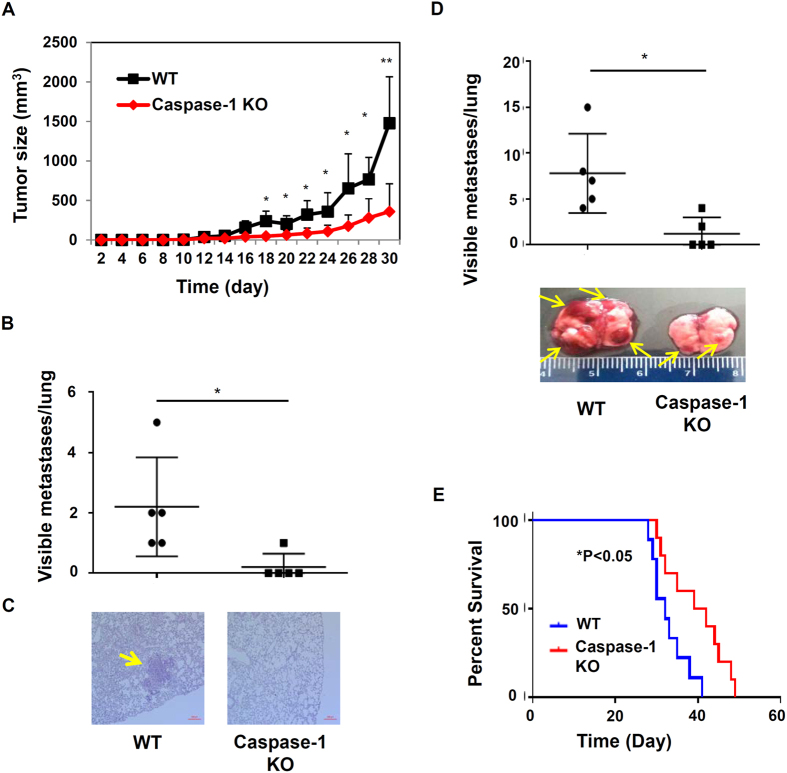
Inflammasome deficient mice exhibit reduced tumor growth and metastasis. (**A**) WT C57BL/6 mice and caspase-1 KO mice (n = 6) were injected orthotopically with 2.5 × 10^5^ EO771 mammary tumor cells. Tumor growth was monitored. (**B**) Four weeks after tumor inoculation, the lungs of tumor-bearing mice were harvested, and the visible tumor nodules were counted. Symbols in scatter plots represent the number of tumor nodules in the lung from individual mouse, and results are expressed as mean ± SD. Data are representative of three independent experiments with similar results. (**C**) Representative H&E staining of lung sections for spontaneous lung metastasis four weeks after tumor inoculation. Scale bars: 200 μm. Arrows mark metastases in lungs. (**D**) WT and caspase-1 KO mice (n = 5) were injected intravenously with EO771 cells. Mice were sacrificed on day 21 post-inoculation. Lungs were harvested, and metastasis nodules were counted. Symbols in scatter plots represent the number of tumor nodules in the lung, and results are expressed as mean ± SD. Lower panel, gross morphology of lungs of WT or mutant mice injected *i.v.* with EO771 cells. Arrows mark visible surface metastases in lungs. *P < 0.05, **P < 0.01 (Student’s *t* test). (**E**) Survival curves of WT and caspase-1 KO mice (n = 6) injected intravenously with 2 × 10^5^ EO771 mammary tumor cells. *P < 0.05, statistical comparisons were performed by two-way ANOVA analysis. Data are representative of three independent experiments with similar results.

To determine the impact of inflammasome activities on tumor progression, we examined mammary tumor growth and metastasis in inflammasome deficient mice. We utilized an orthotopic mammary gland tumor model with EO771 murine breast cancer cells, which are syngeneic to C57BL/6 mice. 2.5 × 10^5^ EO771 tumor cells were injected orthotopically into the 4^th^ fat pads (murine mammary glands) of wild type (WT) and caspase-1 knockout (KO) female mice (all on the C57BL/6 background) at about eight weeks old (Fig. 1), then tumor growth was measured once every two days. In WT mice, tumors grew rapidly two weeks post EO771 injection. However, primary tumor growth in inflammasome deficient mice was significantly reduced (Fig. 1A). When implanted into fat pads of immunocompetent WT mice, EO771 tumor cells also spontaneously develop metastasis in lungs. In our experimental condition, usually 1–5 visual tumor nodules formed spontaneously in the lung of WT mice four weeks after implantation of EO771 cells into fat pads. Notably, caspase-1 KO mice had significantly fewer lung metastases. Accordingly, HE staining of lung tissues showed that caspase-1 KO mice with mammary gland tumors had decreased numbers of tumor foci (Fig. 1B,C).

To further define the role of inflammasome/IL-1 pathways in cancer metastasis, we utilized an experimental metastasis model through *i.v.* injection of EO771 cells. WT and caspase-1 KO mice were injected via tail vein with EO771 cells, then mice survival and lung metastasis were examined. As shown in Fig. 1D, caspase-1 KO mice had reduced lung metastasis three weeks after *i.v.* injection of EO771 cells, as indicated by number of tumor nodules and gross morphology of the lungs. In survival experiments, Kaplan-Meier analysis demonstrated that inflammasome deficient mice had improved survival rate compared with WT mice (Fig. 1E). Those results suggest that inflammasomes in tumor microenvironments support tumor growth and metastases.

### NLRP3 Inflammasome is involved in tumor progression

**Figure 2:**
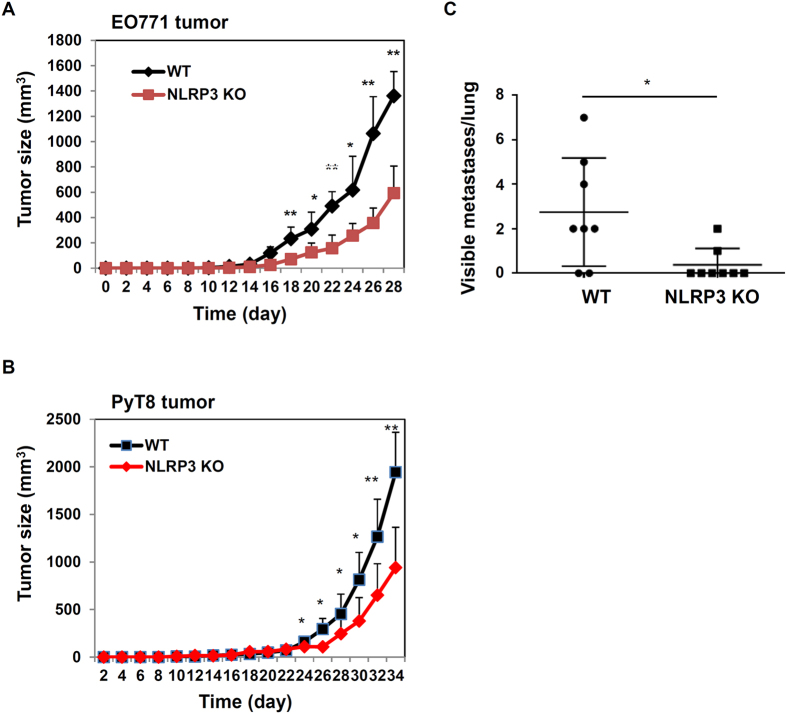
Effects of NLRP3 on tumor growth and metastasis. (**A**) WT and NLRP3 KO mice (n = 6) were injected orthotopically with EO771 mammary tumor cells. Tumor growth was measured. (**B**) and (**C**). PyT8 murine mammary tumor cells were implanted into 4^th^ fat pads of WT and NLRP3 KO female mice (n = 8). Tumor growth and lung metastasis were measured. *P < 0.05, **P < 0.01 (Student’s *t* test). Data are representative of three independent experiments with similar results.

While activation of caspase-1 and secretion of IL-1β can be induced by several NLRP proteins, NLRP3 inflammasome is the most common type, and can be activated by many diversified stimuli^6^,^13^,^18^. Therefore, we investigated whether NLRP3 contributes to tumor growth and metastasis. In the EO771 tumor model, WT and NLRP3 KO female mice were injected orthotopically with tumor cells. As shown in Fig. 2A, NLRP3 deficient mice had reduced tumor growth, which is similar to what we observed in caspase-1 KO mice. To independently verify our results, we used another murine mammary tumor cell line PyT8, which was derived from mammary tumors spontaneously developed in an MMTV-PyMT transgenic mouse in our laboratory. PyT8 cells form tumors when implanted into 4^th^ fat pads of syngeneic C57BL/6 WT female mice. Our results showed that tumor growth and metastasis were also decreased in NLRP3 KO mice after injection of PyT8 tumor cells (Fig. 2B,C). Collectively, those results suggest that the activation of NLRP3 inflammasome promotes tumor growth and metastases in breast cancer orthotopic models.

### Increased IL-1β levels at primary tumor and metastatic sites

**Figure 3:**
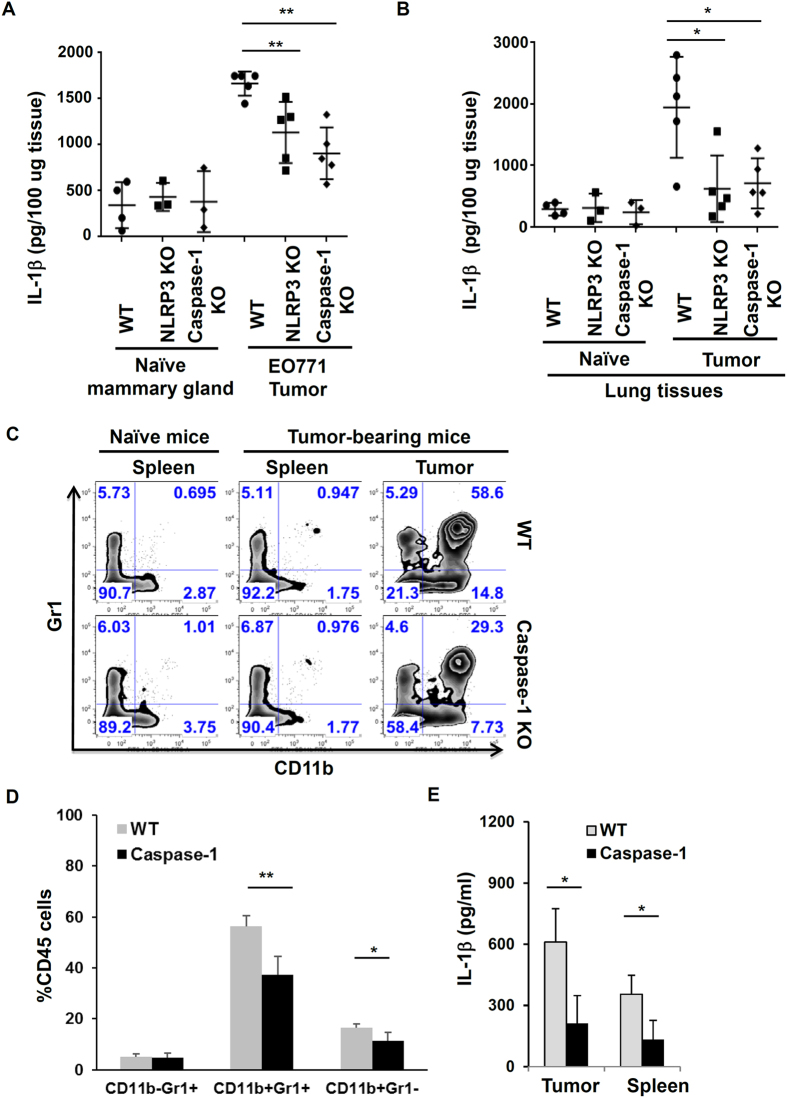
Inflammasome activation is associated with increased IL-1β levels and infiltration of myeloid cells in tumor microenvironments. (**A**,**B**) The levels of IL-1β protein in tissue samples from WT, NLRP3 KO and caspase-1 KO mice (n = 5) injected with EO771 tumor cells. Mammary glands (naïve mice) and tumor tissues and lungs were harvested and homogenized. IL-1β concentration in tissues was measured by ELISA. *P < 0.05, **P < 0.01. (Student’s *t* test). Representative data from one of at least three independent experiments are shown. (**C**) Flow cytometry analysis of myeloid cell populations in spleen and tumor tissues from WT and caspase-1 KO mice injected with EO771 tumor cells. Plots were gated on live and CD45^+^ cells. Numbers indicate percentage of CD11b^+^Gr1^−^, CD11b^+^Gr1^+^, or CD11b^−^Gr1^+^ cells of CD45 cells. (**D**) The Quantification and statistical difference in myeloid cell populations as in 3C (n = 5). Results are shown as mean ± SD. Student’s t test was performed and statistical significance is indicated by *P < 0.05, **P < 0.01. (**E**). IL-1β concentration in culture supernatants of CD11b^+^ cells isolated from spleen and tumor tissues after cultured for 24 hours. *P < 0.05, (Student’s *t* test).

The reduced tumor growth and metastasis in NLRP3 and caspase-1 deficient mice suggest that inflammasome activity influences tumor development. To test whether inflammasome-mediated IL-1β production is linked to tumor progression, we measured IL-1β levels in tumor tissues. Primary tumors from tumor-bearing mice, and normal mammary glands from naïve mice (controls) were collected, homogenized and assayed by IL-1β specific ELISA. We found elevated concentration of IL-1β in tumor microenvironments from WT mice. However, tumor tissues from caspase-1 deficient mice had reduced levels of IL-1β (Fig. 3A). To confirm that IL-1β production is the result of inflammasome activation in the tumor microenvironment, we examined the processing of pro-IL-1β proteins in tumor tissues by Western blot. We found that the amount of mature or processed IL-1β and caspase-1 proteins in tumor tissues of caspase-1 KO and NLRP3 KO mice were significantly decreased, compared to that of WT mice (Supplemental Fig. S1).

Next, we examined inflammasome activities at metastasis sites. Lung tissues from naïve or tumor-bearing mice were homogenized and assayed for mature IL-1β protein production. While naive mice from both groups had similar and very low levels of IL-1β in lung tissues, tumor-bearing WT mice had increased levels of IL-1β protein in the lung (Fig. 3B). In contrast, lung tissues from tumor-bearing caspse-1 KO mice produced less mature IL-1β protein. These results suggest that the activation of inflammasomes and production of IL-1β in primary tumors may create favorable microenvironments for tumor metastasis.

### Inflammasome promotes the recruitment of myeloid cell populations

Our results indicate that the activation of inflammasomes and production of IL-1β *in vivo* provide inflammatory microenvironments to promote tumor growth and metastasis. To investigate how the inflammasome pathway modulates tumor microenvironments, we analyzed infiltration of immune cells in tumor tissues. Single cell suspensions were prepared from tumor tissues and spleen, then analyzed by FACS for phenotypes of myeloid cells. As shown in Fig. 3C,D, myeloid cells (CD11b^+^GR1^−/low^ cells and CD11b^+^Gr1^+^) were significantly increased in tumor tissues in WT mice with EO771 tumors, compared to spleen and normal mammary glands of naïve mice. Generally, CD11b^+^GR1^−/low^ cells are referred to as tumor-associated macrophages (TAMs) and CD11b^+^Gr1^+^ myeloid cells as myeloid-derived suppressor cells (MDSCs), even though they are also heterogeneous populations^38^,^40^,^41^,^42^,^43^,^44^,^45^,^46^,^47^. Importantly, the infiltration of tumor-associated CD11b^+^ cells, including CD11b^+^Gr1^−^ TAMs and CD11b^+^Gr1^+^ MDSCs, was reduced in inflammasome deficient mice. In contrast, CD11b^−^Gr1^+^ neutrophils were comparable between WT and KO mice. Those results show that activation of inflammasomes enhanced the infiltration of myeloid cells, including MDSC and tumor-associated macrophages, into tumor tissues.

To test whether myeloid cells in tumor microenvironments have increased inflammasome activity, CD11b^+^ cells were isolated from tumor tissues, and cultured for 24 hours without any stimulation. As shown in Fig. 3E, CD11b cells from tumor tissues of WT mice produced a considerable amount of IL-1β, but spleen CD11b cells produced a relatively low concentration of IL-1β. Compared to WT cells, CD11b cells from tumor tissues of caspase-1 deficient mice secreted significantly less IL-1β. Because chemokine (C-C motif) ligand 2 (CCL2) is involved in monocytes or macrophages recruitment, we examined the effects of IL-1β on CCL2 levels. Our results show that IL-1β induced the expression of CCL2 in macrophages and tumor cells (Supplemental Fig. S2). However, further studies are required to prove that inflammasome pathways modulate tumor microenvironments through CCL2-mediated recruitment of myeloid cells from the bone marrow.

### Increased IL-1β levels in primary tumor tissues and metastatic sites in a spontaneous murine mammary gland tumor model

**Figure 4:**
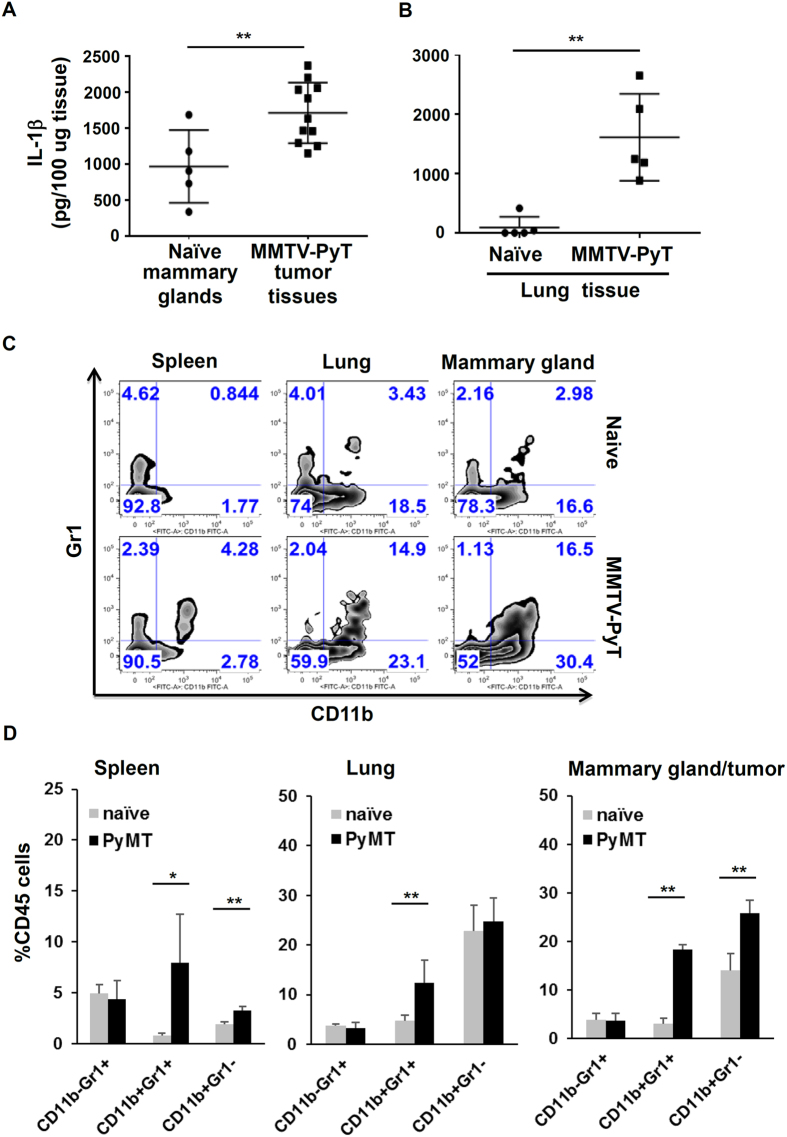
Increased IL-1β levels and myeloid infiltration in primary tumor tissues and metastatic sites in a spontaneous murine mammary gland tumor model. (**A**) Tumor tissues (n = 11) from MMTV-PyMT mice of 12 weeks old, and normal fat pads (n = 5) from littermate controls were isolated and homogenized. The levels of IL-1β protein in tissue samples were measured by ELISA. **P < 0.01. (**B**) IL-1β levels in lung tissue from MMTV-PyMT tumor–bearing mice and control mice (n = 5). **P < 0.01. (**C**) Myeloid cell populations in spleen, lung and tumor tissues from naïve mice, and MMTV-PyMT tumor-bearing mice. Plots were gated on live and CD45^+^ cells. (**D**) The Quantification and statistical difference in myeloid cell accumulation (CD11b^−^Gr1^+^, CD11b^+^Gr1^+^, and CD11b^+^Gr1^−^) as in 4C (n = 5). Results represent pooled data and are shown as mean ± SD. Student’s t test was performed and statistical significance is indicated by *P < 0.05, **P < 0.01.

To determine whether inflammasome activation also occurs during spontaneous tumor development, we utilized MMTV-PyMT mice as a mammary tumor model, in which the Polyoma virus middle T antigen (PyMT) is expressed under the direction of a mouse mammary tumor virus promoter. MMTV-PyMT mice develop spontaneously multifocal mammary gland tumors with lung metastasis, which is comparable to human breast cancer development^48^,^49^. In our experimental conditions, after 12 weeks, the transgenic mice started to develop multiple tumors in mammary glands. Tumor-bearing mice also developed a variable number of metastasis nodules in the lung. When tumor and lung tissues were homogenized and analyzed by ELISA, mature IL-1β levels in primary mammary tumors and metastasis sites in MMTV-PyMT mice were significantly elevated (Fig. 4A,B). Furthermore, we found that CD11b^+^Gr1^+^ and CD11b^+^Gr1^−^ myeloid cell populations were significantly increased in both tumor tissues and spleen of MMTV-PyMT mice (Fig. 4C,D). Those results suggest that spontaneous mammary tumor development in MMTV-PyMT transgenic mice is also associated with inflammasome activation and infiltration of myeloid cells in tumor microenvironments.

### Blocking IL-1R reduces tumor growth and metastasis in the murine mammary gland tumor model

**Figure 5:**
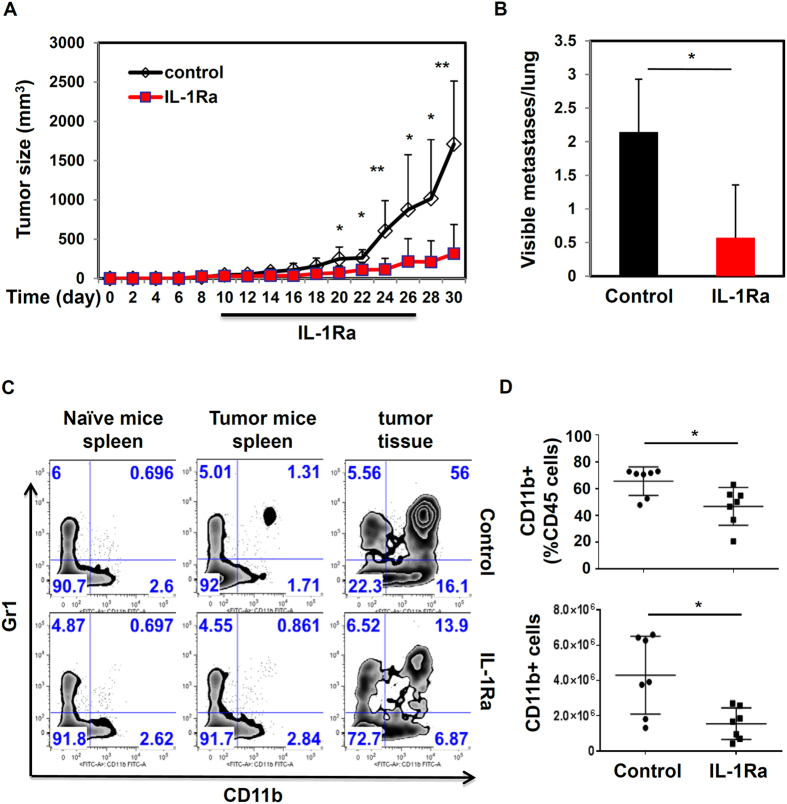
Blocking inflammasome reduces tumor growth and metastasis. (**A**,**B**) 2.5 × 10^5^ EO771 tumor cells were injected into the 4^th^ mammary glands of WT C57BL/6 mice. When tumors reached about 5 mm in size, tumor-bearing mice were injected intraperitoneally with saline (control), or murine IL-1Ra at 5 mg/kg body weight once every two days for two weeks. Tumors were measured with calipers every two days. At the end of experiments, the number of visible tumor nodules per lung was counted. Data represent mean ± SD; *n *= 7 mice per group. (**C**,**D**). The recruitment of myeloid cell populations in spleen and tumor tissues from tumor bearing mice treated with or without IL-1Ra. Plots were gated on live and CD45^+^ cells. Percentage and total numbers of CD11b^+^ cells among CD45^+^ cells are depicted in (**D**). *P < 0.05, **P < 0.01 (Student’s *t* test). Data are representative of three independent experiments.

The reduced tumor growth and metastasis in caspase-1 deficient mice suggest that blocking inflammasome activity or IL-1R signaling may inhibit tumor growth. To test this hypothesis, we utilized IL-1R antagonist (IL-1Ra) to block IL-1 activity *in vivo*. WT mice injected with EO771 cells orthotopically were separated randomly into two groups when tumors reached a size of about 5 mm. Then one group of mice was injected with saline (control), and another group was injected intraperitoneally with murine IL-1Ra at 5 mg/kg once every two days for two weeks. As shown in Fig. 5A, IL-1Ra treatment significantly inhibited tumor growth, compared to untreated mice. Furthermore, tumor-bearing mice treated with the IL-1Ra had a reduced number of tumor nodules in lungs (Fig. 5B). Next, we examined the effects of IL-1R blockade on the infiltration of myeloid cells. As shown in Fig. 5C,D, IL-1R blockade significantly reduced the percentage and total number of CD11b myeloid cells (CD11b^+^Gr1^+^ and CD11b^+^Gr1^−^ cell populations) in tumor tissues. Those results suggest that IL-1 blockade reduces tumor growth and metastasis through modulation of tumor microenvironments.

### IL-1R blockade inhibits human breast cancer progression

**Figure 6:**
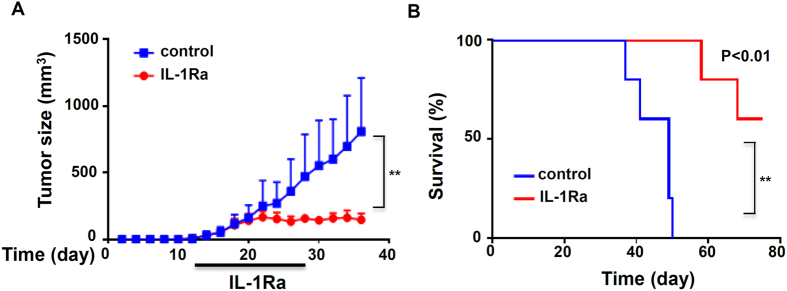
IL-1R blockade inhibits human breast cancer growth in an orthotopic xenograft model. NSG mice were injected orthotopically with 1 × 10^6^ MBA-MD-231 human breast cancer cells. When tumors reached a size of about 5 mm, tumor-bearing mice were injected intraperitoneally with saline, or human IL-1Ra at 5 mg/kg once every two days for two weeks. (**A**) Growth curves and (**B**) Kaplan–Meier survival curves of mice treated with or without IL-1Ra (*n *= 5). **P < 0.01, statistical significance was determined by two-way ANOVA analysis.

To determine whether blocking IL-1R signaling suppresses human breast cancer growth, we utilized an orthotopic xenograft mouse model of human breast cancer cells. MDA-MB-231 cells, a human breast cancer cell line, were injected into fat pads of immunodeficient NSG mice. When tumors reached a size of 5 mm, tumor-bearing mice were treated with human IL-1Ra via *i.p.* injection for two weeks, and tumor growth was measured. As shown in Fig. 6A, IL-1R blockade significantly suppressed the tumor growth of human breast cancer cells in NSG mice. Remarkably, while tumor-bearing NSG mice died because of metastasis, IL-1Ra-treated mice survived much longer (Fig. 6B). This result suggests that targeting the inflammasome/IL-1 pathway leads to reduced tumor growth and metastasis in the xenograft mouse model of human breast cancer cells.

## Discussion

In this study, we demonstrated that inflammasome activation and IL-1β production in tumor-associated macrophages provided an inflammatory microenvironment promoting breast cancer progression. Our results show that tumor growth and metastasis in inflammasome deficient mice were significantly decreased. Primary and metastatic tumor tissues were associated with elevated levels of IL-1β, which potentially induces a number of cytokines, chemokines and transcription factors in both myeloid cells and tumor cells. Inflammasome activation also led to the infiltration of myeloid cells such MDSC and TAMs. Significantly, IL-1 blockade with IL-1R antagonist reduced tumor growth and metastasis in preclinical breast cancer models.

Our results show that inflammasome and IL-1β enhance tumor growth and metastasis in breast cancer models. Currently, the role of inflammasomes in tumor initiation, growth and metastasis remains not fully understood. Many studies about the role of inflammasomes were conducted in the AOM/DSS-induced colon cancer model. Results from those studies indicate that inflammasomes generally provide protection against tumorigenesis because IL-18 is involved in tissue remodeling of the intestine system. For example, Allen *et al*. showed that mice deficient for NLRP3, ASC or caspase-1 had severe colitis and increased tumorigenesis in the AOM/DSS colon cancer model, whereas NLRC4 KO mice did not show any changes in the rate of developing colitis-associated colon cancer^24^. The authors further demonstrated that NLRP3-inflammasome activation in bone marrow-derived cells was critical for the tumor suppression. On the other hand, Hu *et al*. showed that caspase-1 KO and NLRC4 KO mice, but not NLRP3 KO mice, displayed increased tumorigenesis in the same AOM/DSS colon cancer model^27^. In addition, NLRP6 and NLR12 have been shown to suppress tumor development in the AOM/DSS colon cancer model^50^,^51^.

In contrast, other studies revealed that inflammasomes and IL-1β contribute to melanoma and mesothelioma development. Drexler *et al*. found that IL-1R1 KO and caspase-1 KO mice were protected from carcinogen–induced skin cancer^52^. The authors found ASC in different tissues had opposite roles in tumor growth: a tumor-suppressor in keratinocytes, but a tumor-promoter in myeloid cells. Fujita’s group examined the role of ASC in tumorigenesis of human melanoma^53^,^54^. They found that ASC protein levels were downregulated in a group of human metastatic melanoma cell lines compared to primary melanoma cell lines. Van Deventer *et al*. investigated the role of the NLRP3 inflammasome in the immune response induced by a dendritic cell vaccine against the poorly immunogenic melanoma cell line B16-F10. The authors suggested that the expression of NLRP3 in the tumor microenvironment diminishes DC vaccine-induced anti-tumor immunity^55^. However, it is unclear how the inflammasome is activated in melanoma cells. Moreover, several studies suggest that the NLRP3 inflammasome contributed to the development of mesothelioma due to the ability of asbestos to stimulate inflammasome activation^56^,^57^. Based on our studies and published reports, it is likely that inflammasomes can either support or inhibit tumor development depending on tumor models and experimental conditions. Inflammasome-mediated IL-1β can induce the recruitment of TAMs and MDSCs which promote tumor development as reported in this paper. It is also possible that IL-1 directly acts on epithelial or tumor cells to promote proliferation and invasion. On the other hand, inflammasome/IL-1-induced inflammation may induce the activation of adaptive immune cells, enhancing anti-tumor immunity. However, more studies are required to address the involvement and roles of different inflammasomes in tumor progression and tumor immunity.

In the tumor microenvironments, potential danger-associated molecular patterns or tumor-associated molecule patterns can be recognized by Toll-like receptors (TLRs) in innate immune cells. Our results show that tumor development led to the activation of inflammasomes in innate immune cells. However, the molecules trigger the activation of inflammasomes in tumor tissues remains unknown. It is possible that components from dying tumor cells such as ATP, HMGB1, or oxidized DNA induce the activation of NLRP3 inflammasome. Even through this study focused on NLRP3 inflammasome, it does not exclude the possibility that other inflammasomes are involved in tumor growth and metastasis. We speculate that depending on types of tumors or stages of tumor development, various inflammasomes may be activated in tumor microenvironments. The controversial role of inflammasomes in promoting tumor growth and enhancing anti-tumor immunity might be attributed to different NLPRs involved.

Inflammasome activation not only leads to the production of mature IL-1β and IL-18, but also causes cell death termed as pyropoptosis^7^,^9^. Currently, little is known about the role of pyropoptosis in tumor-associated inflammation and immunity. IL-18 has been shown to either promote tumor growth or enhance anti-tumor immunity. Our preliminary data show that tumor growth was not reduced in IL-18 KO mice, indicating that IL-1β and IL-18 may have different or even opposite roles in tumor development (Guo, unpublished results). The IL-1 family has at least 11 members^19^. IL-1α, another IL-1 family member, also binds to IL-1R. While IL-1β is produced mainly by innate immune cells such as macrophages, IL-1α can be secreted by many cell types, including epithelial cells. IL-1α has been suggested to function as an endogenous danger signal due to its release from dying cells. It has been reported that IL-1α and IL-1β may play distinct roles in immune response during infections and inflammatory diseases^58^,^59^. However, further studies are needed regarding the unique function of each IL-1 family protein, including IL-1α, IL-1β, and IL-18, in the tumor development.

Our results suggest that the inflammasome/IL-1 pathway induces the infiltration of myeloid cells into tumor tissues. However, very little is known about the mechanisms responsible for inflammasome-induced myeloid cell recruitment. Since chemokines regulate the recruitment and accumulation of immune cells to infected or inflamed tissues, we think that inflammasome and IL-1 pathways influence the recruitment of myeloid cells through the induction of chemokines^60^,^61^,^62^,^63^. IL-1R transduces signals through Myeloid Differentiation Factor 88 (MyD88), a key adaptor protein for IL-1R and TLRs, which triggers a series of signal events, leading to the activation of both the NF-kB and MAPK pathways. Activation of these pathways results in the expression of inflammatory genes, including chemokines that mediate immune cell recruitment and mobilization^64^,^65^. A number of chemokines, such as CCL2, CCL5 and CXCL12, may contribute to the recruitment of innate immune cells^60^,^61^,^66^,^67^. For example, CCL2 is a member of the C-C chemokine family, which regulates the recruitment of myeloid cells into inflamed sites during pathogen infection. CCL2 has also been shown to regulate the infiltration of macrophages into tumor tissues^66^,^67^,^68^. We found that IL-1β induced CCL2 expression in macrophages and tumor cells (Supplemental Fig. S2). This result implies that inflammasome activation may increase CCL2 production, which promotes myeloid cell recruitment. However, further molecular and animal experiments are needed to test that inflammasome/IL-1β-induced CCL2 regulates the recruitment of macrophages in tumor microenvironments and metastatic sites. As IL-1β is a potent cytokine that can upregulate the expression of many inflammatory cytokines and chemokines. Thus it is possible that the inflammasome/IL-1 pathway may induce the recruitment of various myeloid cells thorough multiple factors.

In summary, this study shows that the inflammasome and IL-1β pathway induces inflammatory microenvironments promoting tumor growth and metastasis. Our results also highlight the role for inflammasome and IL-1β in regulating myeloid cell recruitment. Our study further suggests that targeting the tumor microenvironment through inflammasome/IL-1 blockade may provide a novel approach for cancer treatment.

## Methods

### Reagents

All chemicals used in the present study were purchased from Sigma (St. Louis, MO), unless otherwise noted. Ultrapure LPS, ATP, Caspase-1 inhibitor Ac-YVAD-cmk were purchased from Invivogen (San Diego, CA). Murine or human IL-1β, IL-1α and IL-18 from PeproTech (Rocky Hill, NJ); mouse IL1Ra from Novus Biotech, and human IL-1Ra from PeproTech. Human or murine IL-1β ELISA kits were from R&D system (Minneapolis, MN) or eBioscience (San Diego, California); Antibodies to human IL-1β, human caspase 1, mouse IL-1β and mouse caspase 1 were from R&D Systems or Santa Cruz Biotechnology (Santa Cruz, CA).

### Cell lines and cell culture

Murine mammary tumor cell lines EO771 and PyT8, human breast cancer cell line MDA-MB-231 were cultured in DMEM media supplemented with 10% fetal bovine serum (FBS), 100IU/mL penicillin, 100 mg/mL streptomycin. Primary immune cells including bone-marrow-derived macrophages (BMDMs), spleen cells, and purified CD11b^+^ cells from spleen or tumors were cultured in DMEM media supplemented with 10% heat-inactivated fetal bovine serum (FBS), 100IU/mL penicillin, 100 mg/mL streptomycin.

### Mice and Tumor models

All mice including C57BL/6J, NSG, MMTV-PyMT, caspase-1 KO, NLRP3 KO were purchased from the Jackson Laboratories (Bar Harbor, Maine). All mice were maintained at MUSC Hollings animal facility under specific pathogen-free conditions. ***Breast cancer orthotopic implant model:***EO771 and PyT8 are mammary tumor cell lines that are syngeneic to C57BL/6 mice. 2.5 × 10^5^ EO771 tumor cells, or PyMT mammary tumor cells were injected orthotopically into the 4^th^ mammary glands of WT or mutant female mice at age of 8 weeks old. Tumors were measured bidimensionally with calipers once every two days, and tumor volume was calculated by the formula (length × width × height) × 0.5. At the end of experiments or time indicated otherwise, lungs were harvested, and lung tumor nodules were counted directly. ***Human breast cancer xenograft model:*** Human MDA-MB-231 breast cancer cells (1 × 10^6^ cells) were implanted into 4^th^ fat pads of NSG mice. Tumors were measured bidimensionally with calipers once every two days. ***Experimental metastasis model:*** WT or mutant mice were injected intravenously with 2 × 10^5^ EO771 mammary tumor cells. Mice survival was monitored, and then on day 28 post injection, lungs were harvested, and counted. Lung tissues were fixed for further analysis. ***IL-1R blocking experiments:*** WT mice injected with EO771 cells orthotopically in the fat pads were separated randomly into two groups when tumors reached a size of 5 mm. Then one group were injected with saline (control), and another group of mice was injected intraperitoneally with IL-1Ra at 5 mg/kg once every two days for two weeks. Mice were evaluated for tumor growth and metastasis. All experimental protocols involving mice were approved by the Institutional Animal Care and Use Committee (IACUC) of MUSC. All methods and procedures were conducted in accordance with federal regulations as well as institutional guidelines and regulations.

### Isolation of CD11b^+^ myeloid cells

Tumor-associated macrophages were isolated from tumor tissues of mice injected with tumor cells, or MMTV-PyMT transgenic mice, or NSG mice injected with human breast cancer cell MD-MBA-231. Tissues were minced into small pieces and digested with 1 mg/ml type 1A collagenase (Sigma), and 0.1 mg/ml DNase (Sigma). The digested tissues were filtered through 100 μm and 70 μm cell strainer to obtain single cell suspensions. Mouse CD11b^+^ myeloid cells were isolated and purified by magnetic activated cell sorting with anti-CD11b microbeads (Miltenyi Biotec) according to the manufacturer’s protocol.

### FACS, Western Blot and ELISA

Single cell suspensions were stained with viability dye and conjugated antibodies to surface markers. Samples were acquired on a BD FACSVerse, and data were analyzed with Flowjo software. Cell lysates, cell culture supernatants, or tumor homogenates were analyzed by SDS-PAGE and Western Blot with specific antibodies as described. Cytokine concentration in culture supernatants was determined by cytokine-specific ELISA kit (eBioscience or R&D system) per the manufacturer’s instructions^17^,^69^.

## Additional Information

**How to cite this article**: Guo, B. *et al*. Targeting inflammasome/IL-1 pathways for cancer immunotherapy. *Sci. Rep.* **6**, 36107; doi: 10.1038/srep36107 (2016).

**Publisher’s note:** Springer Nature remains neutral with regard to jurisdictional claims in published maps and institutional affiliations.

## Supplementary Material

Supplementary Information
