# Supplementary material for: Targeting inflammasome/IL-1 pathways for cancer immunotherapy
Source: Sci Rep. 2016 Oct 27;6:36107. doi: 10.1038/srep36107 (PMC5082376; doi:10.1038/srep36107)
Supplement: Supplementary Information [file srep36107-s1.pdf]

Supplementary Information for

Targeting inflammasome/IL-1 pathways for cancer immunotherapy

Beichu Guo [1,2,*], Shunjun Fu[1], Jinyu Zhang[1], Bei Liu[1,2], Zihai Li[1,2]
1 Department of Microbiology and Immunology, 2 Hollings Cancer Center, Medical University of South Carolina (MUSC), Charleston, South Carolina 29425-5040. United States of America.

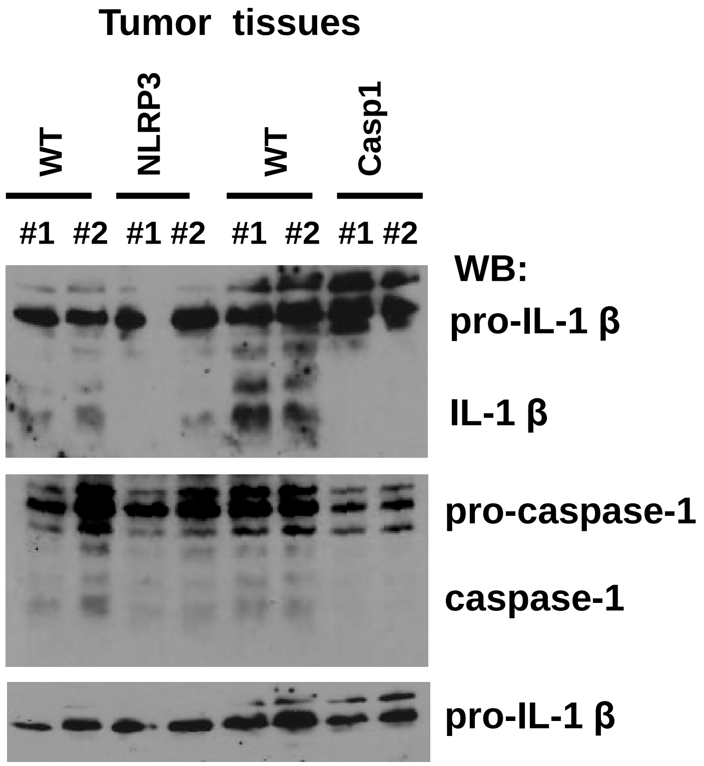

Supplemental Figure S1. Inflammasome activation in tumor microenvironments. Western Blot analysis of tumor tissues from WT, NLRP3 KO and caspase-1 KO mice injected orthotopically with EO771 tumor cells. Tumor tissues were harvested, homogenized, and analyzed by Western Blot with anti-IL-1β or anti-caspase-1 antibodies for caspase 1 and IL-1β processing.

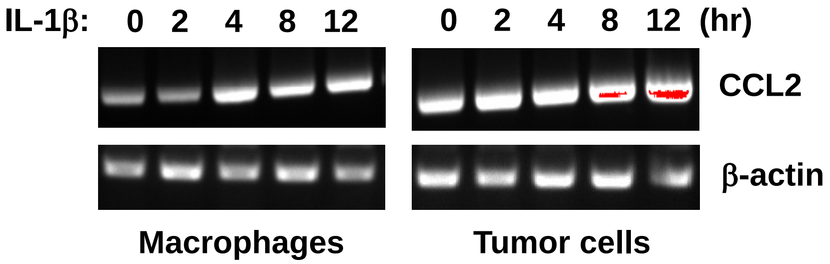

Supplemental Figure S2. IL-1β induces CCL2 expression in macrophages and tumor cells. RT-PCR analysis of CCL2 expression in macrophage cell line RAW 264.7 and breast cancer cell line EO771 treated with IL-1β.
